# Exploring public knowledge and perceptions regarding per os OTC pain-relieving medications: the case of paracetamol (acetaminophen)

**DOI:** 10.1186/s40545-023-00598-1

**Published:** 2023-07-20

**Authors:** Michael Petrides, Aliki Peletidi, Christos Petrou, Evangelia Nena, Maria Papavasili, Theodoros Constantinidis, Christos Kontogiorgis

**Affiliations:** 1 Laboratory of Hygiene and Environmental Protection, Medical School, Democritus University of Thrace, Campus (Dragana) Building 5, 68100 Alexandroupolis, Greece; 2 Pharmacy Program, Department of Health Sciences, School of Life and Health Sciences, University of Nicosia, 2417 Nicosia, Cyprus

**Keywords:** Over-the-counter medications, Pain relief, Paracetamol/acetaminophen/APAP use, Community pharmacies, Pharmacoepidemiological study, Republic of Cyprus

## Abstract

**Background:**

Over-the-counter medications (OTC) are safe and effective when patients follow the patient’s information leaflet (PIL) instructions and/or the instructions given by healthcare professionals (HCPs). However, OTC medications could be harmful and unsafe when individuals do not follow the given instructions and/or when their understanding about the proper use of OTC medications is incorrect. This study aimed to investigate the knowledge and perceptions of people regarding paracetamol use in the Republic of Cyprus.

**Methods:**

This cross-sectional study, which belongs to quantitative research methods, included participants visiting community pharmacies in the following three cities of the Republic of Cyprus: Nicosia, Limassol and Larnaca. Participation in the study was voluntary and anonymous. Participants responded to the survey-based questionnaire, which concerned their knowledge and views on paracetamol use. After the data collection, responses were tabulated and analysed statistically.

**Results:**

The original compound was shown to be more well-known compared to generics. A notable percentage of respondents—ranging between 13.0% (*N* = 49) and 29.8% (*N* = 112)—answered incorrectly that broadly used non-steroidal anti-inflammatory drugs (NSAIDs) contain paracetamol. Furthermore, a remarkable percentage of respondents (71.5%, *N* = 269 and 50.3%, *N* = 189, respectively) falsely believed that two widely used combination products in the market of Cyprus (Paracetamol and Hyoscine-*N*-butylbromide; Paracetamol and Codeine and Caffeine) did not contain paracetamol. A notable percentage of participants (27.6%, *N* = 100) believed that paracetamol causes low toxicity. More than a third of the respondents (40.2%, *N* = 149) drink alcohol together with or slightly after consuming paracetamol products. This viewpoint was linked with the participants’ attitude towards consuming paracetamol medications after drinking alcohol (OR for consuming alcohol versus not consuming alcohol 0.100, 95% CI 0.044–0.225, *p* = 0.000).

**Conclusions:**

To the best of our knowledge, this is the first study conducted in the Republic of Cyprus on this topic. Paracetamol is frequently consumed by individuals, both in its generic and original forms. However, the study showed that respondents often misperceive NSAIDs and paracetamol-containing medications. In addition, it is identified that there is a lack of education among people about the safe and effective use of paracetamol, namely, indications, potential side effects, maximum daily dose, alcohol consumption, and the potential risks of hepatotoxicity. The study contributed to the current published literature as it showed that there is a significant public health issue, for which appropriate measures can be established by the respective Authorities of Cyprus.

**Supplementary Information:**

The online version contains supplementary material available at 10.1186/s40545-023-00598-1.

## Background

Over-the-counter (OTC) medications are widely used to treat minor ailments globally [1, 2]. The use of OTC helps both individuals and National Health Systems (NHS) to lower their costs worldwide, as it can reduce doctor visits, leading to less expensive medical treatments [3]. Paracetamol (a non-opioid painkiller) is one of the most frequently used analgesic/antipyretic medication sold either as an OTC or as a prescription-only medicine (POM) internationally [4, 5]. It can be bought as a single drug or as a combination drug formulation. Indications for its use include mild to severe musculoskeletal pain, fever, nasal congestion, migraines, etc. [6].

In Cyprus, paracetamol-containing products are authorised by the Pharmacy Board [7] mainly through the National Procedure [8–10]. According to the latest Ministerial Decree (KDP 465/2017), which defines whether a medicinal product is subject to medical description (with or without renewal), paracetamol is *“subject to medical prescription which may be renewed”* [11]. It can also be dispensed as an OTC medication both for rectal and oral administration (maximum single oral dose: 1 g). Paracetamol is a pharmacy-distributed-only medicine, as it is not included in the relevant general sales list.

Interestingly, the new National Healthcare System in Cyprus (single-payer system) launched in 2019 (named as General Healthcare System—GHS), only one and a half years before the study’s initiation. Therefore, the pharmaceutical market of the Republic of Cyprus is unique in the EU context [12]. Before the economic crisis in Cyprus, overprescribing and overconsumption of medications were the case, with no volume-control measures in place [13]. In addition, irrational prescribing problems, provision and use of both prescription-only medicines and OTC medications, and the subsequent impact on patients’ safety, appear to be more severe in countries without a well-established healthcare under a newly developed national healthcare system [14, 15].

In general, irrational prescribing, provision and use of OTC medications is a common phenomenon in Southern Europe (e.g., Cyprus, France, Greece, Malta and Turkey) [16], especially in countries without a well-organised (differences regarding the structure and process of primary care) [17] or newly developed primary care systems [13]. Moreover, both former and recent studies [18, 19] question the safety of paracetamol use regarding the reported adverse events, as well as both patients’ and pharmacists’ knowledge about paracetamol’s safety and efficacy [20–23]. In addition, previously published literature [23–28] links the intentional (suicide attempts) and the unintentional paracetamol overdose (e.g., accidental use of a paracetamol product with a combination product, such as a per os decongestant, where paracetamol is also included and the patient did not realise that) with both hepatotoxicity and acute liver failure [23–28] but also hepatotoxicity within therapeutic range [29].

Based on the aforementioned, it is apparent that little is known about the appropriate paracetamol use in Cyprus and there is a dire need to narrow this gap. Therefore, our research question was to identify the public’s perceptions and habits about the use of paracetamol-containing medications in the Republic of Cyprus. The choice of the country was primarily based on convenience due to local knowledge, contacts, and ease of collaboration, and on the fact that no literature has been found to date about this important public health issue.

## Methods

This was a cross-sectional study (a type of observational study design) with a quantitative methodological approach. It implemented a survey-based questionnaire, which was already used in a previously published study by Kontogiorgis et al. [21], to examine public knowledge and views of patients that use OTC paracetamol-containing medications (oral route). The data collection tool was validated in Greece (a country with similar behavioural characteristics and attitudes). A quantitative methodology was applied, since it allowed the measurement of quantity through the exploration of numeric patterns. This method is supported by a philosophical approach under the empiricism perspective, a philosophy that assumes knowledge is grounded in what you can see, hear, or experience [30].

### Study population and location

The study was conducted in the three largest cities of the Republic of Cyprus (Nicosia, Limassol, and Larnaca). The cities were chosen based on their population and on the researchers’ convenience.

The chosen cities have approximately 719,000 inhabitants (856,960 total population according to the 2011 census) [31]. For a total period of 6 months, the study’s researchers approached members of the public who visited community pharmacies to pursue their medications (either prescribed or OTC medications). Researchers gave them details about the scope of the study. Participants gave their indirect consent upon the questionnaire’s completion. The exclusion criteria were: (i) age < 18 years, (ii) reluctance to participate, (iii) mental handicap and (iv) incompetence to communicate in the Greek language. The calculated sample size was 377 participants based on the population size of the three selected cities of the Republic of Cyprus. The sample size was calculated using Raosoft online sample calculator [32, 33]. The calculation was based on 50% response distribution, 5% margin of error and 95% confidence interval.

### Questionnaire and data collection

Individuals answered the multiple choice survey questions anonymously. The questionnaire has also been translated into English (Additional file 1). The questionnaire was developed after an extensive literature search by the study’s investigators [18]. The questionnaire contained 18 questions and it was divided into two sections, including demographics. Topics covered participants’ perceptions and knowledge about which medications contain paracetamol, the frequency of paracetamol use and the common reasons for their use, the usual dosage scheme, the maximum daily dose, the concomitant use of paracetamol and alcohol, their knowledge of paracetamol’s toxicity, etc.

The completion time was approximately 15 min. The data collection was conducted in person by giving a hard-copy questionnaire to each of the participants. It took place between November 2020 and April 2021. Before data collection, a pilot study was performed with three pharmacists (one practicing in each of the chosen cities) to enhance the rigour and validity of the study, with no major changes recommended.

### Data management, documentation and curation

#### Managing, storing and curating data

Data at the University of Nicosia fileservers are secure, redundant, and multi-site server located. Data are backed up daily onto secure servers in the host institution. To aid collaboration an Office 365 Team space was created. It is a collaborative space for storing, sharing, and working on digital information. It enabled researchers from all sites to share and view files in a secure 1 TB central repository through a web interface and reduce the need to replicate files across sites.

### Metadata standards and data documentation

Filenames identified clearly and uniquely each data set. The study’s data were stored with a description of the experimental protocol and any events that might have influenced the data.

### Data preservation strategy and standards

Raw data will be retained 2 years after the project ends on secure local servers at the host institution. Analysed data and metadata will be made available after publication.

### Data analysis

Both descriptive and inferential statistical analyses were performed. The Chi-square test, a non-parametric statistic, was used to identify proportional differences between subgroups to assess the determining factors for correct knowledge and attitude towards paracetamol use. Statistical analysis (Crosstab analysis, Odds ratio) was performed using Statistical Package for the Social Sciences (SPSS Inc., Chicago, IL, USA) for Windows (28.0 software version) [34].

### Ethics approval

The study and all relevant documentation, including the survey-based questionnaire, were ethically approved before data collection by the Cyprus National Bioethics Committee (No. EEBK ΕΠ 2020.01.212).

## Results

### General characteristics of participants

In total, 454 individuals were approached. However, 375 individuals (response rate of 82.5%) participated voluntarily in the study by completing the questionnaire. Participants’ mean age was 40.5 years (standard deviation ± 13.7 years), 179 (47.7%) were males and 196 (52.3%) were females. With regard to their educational level, almost half of the sample were high school graduates 40.3% (*N* = 140), whereas 11.8% (*N* = 41) held an M.Sc. or a Ph.D. and 5.5% (*N* = 19) were healthcare professionals (HCPs). The characteristics of the participants are shown in Table 1**.**

**Table 1:** General characteristics of the participants

Characteristics						
Total number of participants (response rate)			375 (82.5%)			
Gender (%)			%			
Male			47.7 (*N* = 179)			
Female			52.3 (*N* = 196)			
Age, years (mean ± standard deviation)						
All participants			40.5 ± 13.7			
Male			43.8 ± 13.2			
Female			37.5 ± 13.5			
Age group, years	%	*N*	%	*N*	%	*N*
	(All)		(Males)		(Females)	
18–29	22.9	86	14.5	26	30.6	60
30–39	29.0	109	26.8	48	31.1	61
40–49	21.3	80	24.6	44	18.4	36
50–59	17.0	63	21.2	38	12.8	25
60–69	8.0	30	10.6	19	5.6	11
> 70	1.9	7	2.2	14	1.5	3
Educational background (%)						% (*Ν*)
Middle school graduate (9 years)						9.0 (31)
High school graduate (12 years)						40.2 (139)
University graduate (16–17 years)						33.5 (116)
Postgraduate or PhD						11.8 (41)
Healthcare professional (physician, dentist, pharmacist, or nurse)						5.5 (19)

#### Views and perceptions of the public about the use of paracetamol

Initially, participants were asked to identify and choose which of the medications contained paracetamol (alone or in combination). More specifically, they had to select among a list of eight commonly used analgesic medications: (a) three contained exclusively paracetamol as the active compound, (b) one was classified as antispasmodic (combined with paracetamol), (c) three were NSAIDs, and (d) one was a weak opioid (combined with paracetamol and caffeine). The participants’ answers are shown in Table 2.

**Table 2:** Active compounds and trade names of the included medications (both original and generics)

Active compounds (plus trade names®)	Participants believing that the active compound is paracetamol (%)
	All participants % (*N*)
Paracetamol	
Original (Panadol®)	87.2 (328)
Generic 1 (Depon®)	50.8 (191)
Generic 2 (Apotel®)	14.1 (53)
Paracetamol + Codeine + Caffeine (Solpadeine®)	49.7 (187)
NSAIDs	
Mefenamic acid (original—Ponstan®)	29.8 (112)
Acetyl-salicylic acid (original—Aspirin®)	25.8 (97)
Nimesulide (generic—Nimm®)	5.6 (21)
Diclofenac (original—Voltaren®)	13.0 (49)
Antispasmodics	
Hyoscine-*N*-butylbromide + Paracetamol (original—Buscopan plus®)	28.5 (107)

*NSAIDs* non-steroidal anti-inflammatory drugs

Logistic regression analysis revealed that neither the gender nor participants’ age was a factor that could predict the correct answers for all questions about medications containing paracetamol. However, the higher the level of education was associated with the higher probability of answering all questions regarding paracetamol-containing drugs correctly (OR 0.111, 95% CI 0.025–0.487, *p* = 0.000) [middle and high school graduates versus university graduates, postgraduates or PhD and HCPs].

In addition, the questions concerning each one of the listed medications were analysed separately. The questionnaire included some of the most popular OTC medications in the Republic of Cyprus. The original paracetamol medication was highly identified by participants (87.2% *N* = 328) regardless of their educational level, age, or gender.

One of the generic paracetamol medications (Depon®) was identified only by 50.8% (*N* = 191) of the participants, regardless of their age. A statistically significant association between gender and knowledge was observed in the responses regarding this specific medication (OR for female versus male 0.554, 95% CI 0.368–0.835, *p* = 0.003), with males having a higher probability of giving incorrect answers than females (Table 3). Similarly, there was a statistically significant association between education and knowledge in the responses regarding this specific medication (OR for lower education versus higher education 1.805, 95% CI 1.179–2.764, *p* = 0.004), with participants of higher education (university graduates, postgraduates or PhD, and HCPs) having a higher probability of giving the correct answer than participants of lower education (middle and high school graduates) (Table 3).

**Table 3:** Logistic regression analysis of the factors predicting the correct answer to each question with a statistical significance

Medication/factors	OR	95% CI	*p* value
Paracetamol			
Original product			
Gender^a^	0.676	0.367–1.244	0.133
Generic product 1			
Gender^a^	0.554	0.368–0.835	0.003
Education^b^	1.805	1.179–2.764	0.004
Generic product 2			
Gender^a^	0.892	0.498–1.598	0.407
Nimesulide (generic product)			
Gender^a^	0.529	0.209–1.342	0.128
Paracetamol + Codeine + Caffeine (original product)			
Gender^a^	0.927	0.618–1.390	0.395
Mefenamic acid (original product)			
Gender^a^	0.697	0.445–1.091	0.071
Acetyl-salicylic acid (original product)			
Gender^a^	0.571	0.355–0.918	0.013
Diclofenac sodium (original product)			
Gender^a^	0.619	0.332–1.154	0.086
Paracetamol + Hyoscine-*N*-butylbromide (original product)			
Gender^a^	0.632	0.400–0.998	0.031

*OR* odds ratio, *CI* confidence interval

^a^Females versus males

^b^Better educated (university graduates, postgraduates or PhD, and health care professionals) versus less educated (middle school graduates and high school graduates)

Nevertheless, only 14.1% (*N* = 53) of the participants identified the second generic paracetamol medication (Apotel®) as a paracetamol-containing one, regardless of their education level, age, or gender (Table 2).

A medication that contains mefenamic acid (Ponstan®) is well-known and used as an analgesic drug. However, 29.8% (*N* = 112) of the participants falsely answered that it contained paracetamol, regardless of their educational level, age or gender.

Nearly one in four (25.8%, *N* = 97) participants falsely considered the very popular acetyl-salicylic acid medication (Aspirin®) as a paracetamol-containing medication. A statistically significant association between gender and knowledge was identified in the responses regarding this specific medication (OR for female versus male 0.571, 95% CI 0.355–0.918, *p* = 0.013), with males having a higher probability of giving incorrect answers than females (Table 3).

A low but notable percentage of the participants (13.0%, *N* = 49) falsely believed that a well-known diclofenac medication (Voltaren®) contained paracetamol, regardless of their educational level, age or gender. This percentage is similar to the participants that did not know that the original paracetamol medication contains paracetamol (12.8%, *N* = 48).

A significant percentage of the participants (71.5%, *N* = 269) did not know that a very common antispasmodic/analgesic medication (Buscopan-plus®) also contained paracetamol, posing a significant danger of consuming other paracetamol-containing medications simultaneously and thus reaching toxic concentrations of paracetamol. A statistically significant association between gender and knowledge was observed in the responses regarding this specific medication (OR for female versus male 0.632, 95% CI 0.400–0.998, *p* = 0.031), with female participants demonstrating better knowledge than males (Table 3).

The medication that combines paracetamol + codeine + caffeine (Solpadeine®) is quite popular in the Republic of Cyprus market. In fact, it is distributed as an OTC due to the low codeine content per unit dose. Interestingly, half of the participants (50.3%, *N* = 189) did not recognise this medication as a paracetamol-containing one. This false belief was not correlated with the participants’ educational level, age or gender.

The main reported reason for paracetamol use was headache (57.7%, *N* = 202), whereas arthralgia, abdominal pain, cold and flu, and fever were followed (Table 4). Gender was not a significant factor in determining the main reasons for using paracetamol. For both male and female participants, the reported reasons for using paracetamol-containing medications were quite similar. Age, gender, and education were examined, and they were not statistically linked with the reasons for using paracetamol.

**Table 4:** Most common reasons reported by participants for using a paracetamol-containing medication

Reasons	All participants					
	Total		Males		Females	
	%	*N*	%	*N*	%	*N*
Headache	57.7	202	59.7	98	56.2	104
Cold and flu	15.4	54	15.9	26	15.1	28
Arthralgia/abdominal pain	12.6	43	11.6	19	13.0	24
Fever	5.7	20	4.9	8	6.5	12
Dizziness	4.3	15	4.9	8	3.8	7
Other	4.3	15	3.0	5	5.4	10

**Fig. 1:**
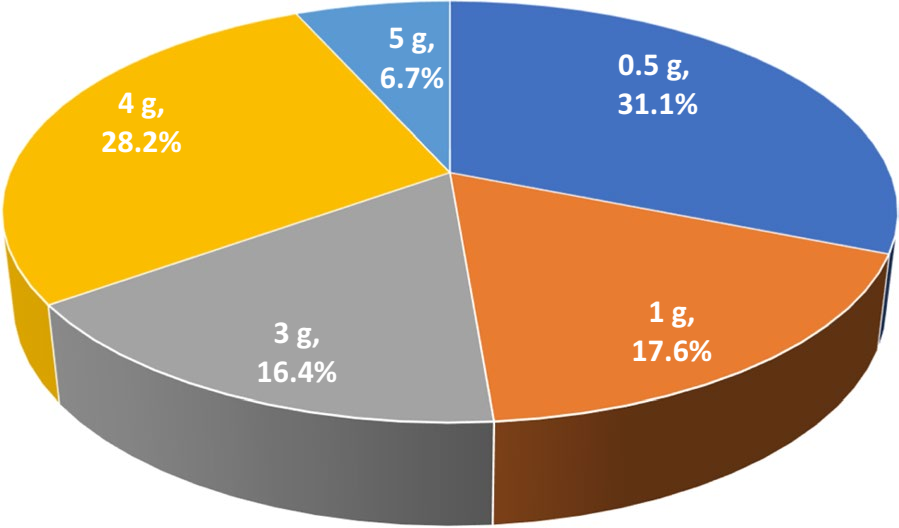
Participants’ responses regarding the maximum daily dose of paracetamol

Regarding the maximum daily dose, only 28.2% (*N* = 96) of the participants answered correctly (4 g). A total of 48.7% (*N* = 166) believed that the maximum daily dose of paracetamol was 1–2 tablets of 500 mg (0.5–1 g), whereas 16.4% (*N* = 56) answered that the total daily dose was 3 g. A notable percentage of participants believed that 5 g was the maximum daily dose (6.7%, *N* = 23), which is toxic (Fig. 1).

A more thorough statistical analysis of the subpopulation that answered 5 g as the maximum daily dose was conducted, revealing a gender linkage. More specifically, 21.7% (*N* = 5) of those participants were females, whereas 78.3% (*N* = 18) were males. A statistically significant association was identified between gender and giving the mistaken answer of 5 g of paracetamol (OR for females versus males 0.223, 95% CI 0.081–0.614, *p* = 0.002), with female participants demonstrating better knowledge than males.

Most of the participants believed that paracetamol-containing medications are toxic to the human body (72.4%, *Ν* = 262), whereas 27.6% (*Ν* = 100) of the sample answered that paracetamol-containing medications are not toxic. The participants’ beliefs on the toxicity of paracetamol were linked with the parallel alcohol consumption with paracetamol medication (OR for parallel alcohol consumption versus no alcohol consumption 0.340, 95% CI 0.166–0.699, *p* = 0.003).

Surprisingly, more than half (58.1%, *Ν* = 200) of the participants answered that they would buy paracetamol medications from places other than pharmacies, whereas a total of 41.9% (*Ν* = 144) answered that they would not buy, either because they would like an expert’s opinion or because they were not sure about the appropriateness of the storage conditions.

#### Source of suggestion and information

Although paracetamol-containing medications do not need a prescription, since they are considered OTC drugs, it is important to examine who suggested their usage to the participants. An outstanding percentage of participants (75.6%, *Ν* = 272) answered that the use of paracetamol-containing medications was suggested to them by a healthcare professional (e.g., a doctor or previously suggested by a doctor or a pharmacist). Some participants answered that they have been advised by a relative to use a paracetamol-containing medication (15.3%, *N* = 55), whereas 9.2% (*N* = 33) answered that they used it after a friend’s suggestion (Table 5). It is good to note that participants who were HCPs stated that the use of a paracetamol-containing medication was suggested by an expert.

**Table 5:** Participants’ responses regarding the source of suggestion for paracetamol use

Source of suggestion	All participants (%) (*N*)
Doctor	18.1 (65)
Previously suggested by a doctor (different occasion)	26.1 (94)
Pharmacist	31.4 (113)
Relative	15.3 (55)
Friend	9.2 (33)

A statistically significant association was identified between participants’ source of suggestion and the source of information, regarding the use of paracetamol medication (OR for suggestion by an HCP versus suggestion by a relative or a friend 5.569, 95% CI 3.161–9.814, *p* = 0.000). Furthermore, a statistically significant association was observed between the participants’ source of suggestion and whether patients with chronic conditions informed their pharmacist before the paracetamol-containing medication use (OR for suggestion by an HCP versus suggestion by a relative or a friend 0.465, 95% CI 0.266–0.812, *p* = 0.006).

The participants were also asked about their source of information about paracetamol-containing medications. Almost half of the participants (48.5%, *Ν* = 160) answered that they were informed by their pharmacist, whereas 24.8% (*Ν* = 82) answered that they were informed by their doctor. A notable percentage (13.0%, *Ν* = 43) answered that they were informed by friends and relatives, leaving the internet (use of websites) (7.3%, *Ν* = 24) and advertisements on both television and Internet (6.4%, *Ν* = 21) as their last sources of information.

#### Concurrent use of paracetamol-containing medications and alcohol

The data collection tool included a set of two questions that aimed to clarify the participants’ concurrent alcohol consumption together with the use of paracetamol-containing medications. First, participants were asked if they consume alcohol after taking a paracetamol-containing medication; more than half responded that they never consume alcohol after paracetamol consumption (59.7%, *Ν* = 221), while some answered that they drink alcohol 2–3 h after paracetamol consumption (27.8%, *Ν* = 103). A notable percentage of volunteers (12.4%, *Ν* = 46) answered that they consumed alcohol after paracetamol consumption, because *“it did not matter”*. This attitude was linked with (a) the participants’ views on paracetamol toxicity (as mentioned before), and (b) the participants’ attitude towards consuming paracetamol medication after drinking alcohol (OR for consuming alcohol versus not consuming alcohol 0.100, 95% CI 0.044–0.225, *p* = 0.000). Second, participants were asked whether they take paracetamol-containing medications, while they already drunk alcohol; the majority of the participants (77.9%, *Ν* = 289) answered that they never took paracetamol-containing medications after alcohol consumption, whereas 22.1% (*N* = 82) answered that they took paracetamol-containing medications after alcohol consumption. Education was linked with this attitude (OR for lower education versus higher education 0.549, 95% CI 0.322–0.936, *p* = 0.018), with participants of higher education (university graduates, postgraduates or PhD, and HCPs) having a higher probability of consuming paracetamol medications after alcohol consumption than participants of lower education (middle and high school graduates).

## Discussion

Paracetamol is a widely used analgesic globally, thus its safety, side effects and interactions are of great concern [4, 5, 18, 28]. To our knowledge, this is the first and only study conducted in the Republic of Cyprus on this topic. Interestingly, most of the current study’s outcomes are in line with a previous study of our research group, conducted in Greece [21]. More specifically, topics include individuals’ recognition of medications containing paracetamol, the main reason for paracetamol use, relatives’ and friends’ advice related to paracetamol use, the maximum allowed dose of paracetamol, whether paracetamol has any side effects or not, the willingness of individuals to buy paracetamol products from non-pharmacy premises or not, and whether alcohol was consumed before or after paracetamol intake.

The most important factors that lead paracetamol to be one of the top self-care medications in adults include (a) the easy access to paracetamol-containing medications in almost 550 pharmacies across the Republic of Cyprus, (b) the availability of paracetamol through the public sector too (i.e., hospital pharmacies) in the recent past, (c) the lack of knowledge regarding the side effects and the toxicity of paracetamol, and (d) the false belief that paracetamol is a safe medication without causing any harmful actions.

### Product identification, paracetamol synonyms, and confusion with NSAIDS

There seems to be a knowledge gap regarding both paracetamol’s recognition (Table 2) and paracetamol’s synonyms [35–38] [i.e., acetaminophen and *N*-acetyl-para-aminophenol (APAP), etc.], which are commonly written on the medicines’ packages and labelling. The aforementioned factors could lead to an unintended overdose of paracetamol-containing medications using them (two or more at the same time) simultaneously (especially products containing more than one active compound).

Moreover, the current study identified that there is a lack of identification of combination products (i.e., 1st product: Paracetamol + Codeine + Caffeine, 2nd product: Paracetamol + Hyoscine-*N*-butylbromide). Specifically, it was found that more than half of the participants [50.3%, *N* = 189 (1st product) and 71.5%, *N* = 269 (2nd product), respectively] did not know that they included paracetamol. According to the Food and Drug Administration (FDA), this is one of the reasons why patients end up taking both OTC paracetamol products and prescribed paracetamol products, resulting in accidental paracetamol overdose (above the maximum daily dosage of 4 g), which could lead to emergency room visits and hospitalisations or it could cause serious liver injury—even death [39]. Data from 1998 to 2003 showed that paracetamol was the leading cause of acute liver failure in the USA, with 48% of paracetamol-related cases (131 of 275) associated with accidental overdose [37]. Besides that, data from 2005 to 2007 also indicated that paracetamol is the almost exclusive cause of liver transplantation related to acute drug overdose, and it represents one-sixth of all-cause acute liver failure transplantation in 7 European countries (France, Greece, Ireland, Italy, the Netherlands, Portugal, UK) [40].

In addition, people often confuse paracetamol with other painkillers including NSAIDs (e.g., aspirin, ibuprofen, etc.) and they are not aware of whether a pharmaceutical product contains paracetamol or not [36–38]. The current study indicated that Cypriot consumers confuse paracetamol-containing medications with NSAIDs, since they have difficulty in recognising which OTC medication contains paracetamol. In few cases, this was associated with consumers’ gender, with women presenting better knowledge.

### Taking analgesics without prescription

According to Lionis et al. study [16], there is a tendency among primary care patients to often exchange OTC medications with friends and family, with no prior consultation by a doctor or a pharmacist in Greece. Current study’s data support that Greece and Cyprus are quite similar regarding the provision of primary care services. Thus, it is not a surprise that some results of this study are in line with a similar study conducted in Greece [21], e.g., the main reason for paracetamol use was to treat headache (GR study 46.8% versus CY study 57.7%), the patients' preference to optionally buy paracetamol-containing products from a non-pharmacy premise (GR study 54.4% versus CY 58.1%), etc.

In the current study, 31.4% of the participants answered that their pharmacist suggested the use of paracetamol, which is higher than the percentage found in Greece (24.5%), showing that there is a potentially higher trust in pharmacists from the patients’ side in the Republic of Cyprus.

It was also found that 13% (*N* = 43) of the participants used paracetamol after their relatives’ or friends’ suggestion, without a previous conversation with a physician or a pharmacist. This is worrying, because this category of patients is at a higher risk of misusing or overusing paracetamol, without knowing its side effects and harmful actions, since they falsely consider paracetamol as a completely safe medication. This leads to the assumption that this patient group might not be aware (without a proper consultation with a doctor or pharmacist before paracetamol use) of how to recognise paracetamol’s side effects and toxicity or what they should do, if an unwanted event occurred.

### False sense of safety—maximum daily dose (MDD)

Previously published literature, showed that consumers perceived paracetamol as a safe medication to use, without recognising the broad range of serious side-effects that paracetamol causes [21, 35, 36, 38, 41–47]. This false sense of security and the lack of knowledge regarding the maximum daily dose of paracetamol can be also observed in the participants’ responses, where 27.6% answered that paracetamol-containing medications are not toxic, and 6.7% (Fig. 1) falsely answered that the maximum daily dose is 5 g (whereas the correct is 4 g), which was gender-related.

The lack of knowledge of paracetamol’s MDD was found in previous studies too [21, 35–37, 41, 42, 45–48] and is one of the most alarming findings, regarding public health. According to the Vordenberg et al. systematic review [41], factors affecting the risk of exceeding MDD include the lack of education, severe pain and/or recurrent pain.

### False sense of safety—paracetamol and concurrent use of alcohol

Disappointedly, the study also identified that there is a lack of knowledge in terms of the concomitant use of alcohol and paracetamol-containing medications, since some individuals might be more prone to liver injury caused by paracetamol use, or those who consume alcohol systematically or have an established liver disease—according to the FDA [28]. A notable percentage of the study’s participants stated that they drink alcohol after paracetamol consumption, because *“it does not matter”* (12.4%). Furthermore, 22.1% (*N* = 82) of the participants responded that they take paracetamol after alcohol consumption (22.1%).

### Restrictions of paracetamol: do they work?

Based on the study’s findings, the patients’ reported use of paracetamol products may lead to safety issues, which were also identified in other countries. This led to the implementation of various and different kinds of restrictions globally, as discussed below.

The FDA established that all medications containing paracetamol in combination with other drugs should not exceed 325 mg of paracetamol per tablet [39]. On the other hand, the European Medicines Agency (EMA) recommended suspending the marketing of modified or prolonged-release products containing paracetamol (alone or combined with tramadol) [49], since *“overdoses with modified-release paracetamol products can be unpredictable in their pharmacokinetics, and complex to manage”* [49]. The recommendation was made by the Pharmacovigilance Risk Assessment Committee (PRAC), which was finally sent to the European Commission which issued an EU-wide legally binding decision.

Furthermore, some European countries implemented pack-size restrictions on pharmacy sales of paracetamol-containing products and/or withdrew sales of paracetamol-containing products from non-pharmacy premises [50]. In addition, in 2011 an 18-year age restriction was implemented in Denmark [51], whereas in Canada [52], England and Wales [53] further initiatives were taken [e.g., warnings of the dangers of paracetamol (both on the packs and the leaflets) as well as package configurations] to communicate the risks of overdose and to enhance product identification and safe use. In Denmark, the age and pack size restrictions were related to a reduction in paracetamol poisonings without establishing a causal link [51]. In Canada, changing the labelling standards for paracetamol-containing products did not manage to reduce hospital admissions due to accidental paracetamol overdose [52]. Moreover, in England and Wales, pack-size restrictions had an unclear contribution to the observed reduction of mortality rates and hospital admissions due to paracetamol toxicity [53]. Interestingly, a reduction was observed in paracetamol-related enquiries to Poison Information Centers in countries, where paracetamol was only available on pharmacy premises [50].

## Limitations, implication, and scope of the study

The scope of the study was to investigate the knowledge and habits of people regarding paracetamol use in the Republic of Cyprus. The main strength of this research is that there have been no other published studies that identified public knowledge and perceptions regarding paracetamol use in the Republic of Cyprus.

It is identified that there is a lack of education among people about the safe and effective use of paracetamol, namely, indications, potential side effects, maximum daily dose, alcohol consumption, and the potential risks of hepatotoxicity. The study contributed to the current published literature as it showed that there is a significant public health issue, for which appropriate measures can be established by the respective Authorities of Cyprus.

The main limitation of the study was that the population was relatively small (*N* = 375) and the findings may be difficult to be generalised. Other limitations include the limited data collection period, the chosen locations (urban areas only and three cities out of four in total in the Republic of Cyprus) and the fact that there were participants who did not complete certain questions. Moreover, there were individuals who did not participate in the study due to the lack of time and/or interest.

## Conclusions

Although paracetamol-containing medications are both widely used and very effective, and in most cases, they do not lead to toxicity, there are also times when a liver injury occurs and might be lethal. Consequently, there are specific issues that need to be managed regarding the OTC use of paracetamol-containing products to safeguard public health. It seems that patients should be educated and counselled more thoroughly by HCPs (mainly pharmacists as they are the first point of call) on the safe and effective use of paracetamol, its indications, potential side effects, maximum daily dose, alcohol consumption, and the risk of hepatotoxicity. Public educational campaigns are useful and should be implemented to emphasise the side effects of paracetamol and to educate the public about paracetamol use only after a previous consultation with an HCP—like all other medications. Community pharmacists are well-positioned to support and educate the public about the safe use of paracetamol and to prevent unintentional harm. Future actions should be developed by the Cypriot Pharmaceutical Association and the pharmacists under their jurisdiction to increase public awareness and knowledge related to paracetamol use. Finally, a further study could be conducted to identify whether public knowledge and behaviours lead to unintended overdose and hospitalisation in Cyprus.

## Supplementary Information

**Additional file 1: Appendix S1.** A translated version of the given questionnaire (the original questionnaire was in Greek language).

## Data Availability

The data that support the findings of this study are available from the corresponding author upon reasonable request.

## Abbreviations

OTC: Over the counter
PIL: Patient’s information leaflet
HCPs: Health care professionals
NSAIDs: Non-steroidal anti-inflammatory drugs
APAP: *N*-Acetyl-para-aminophenol
NHS: National Health Systems
Ph.D.: Doctor of Philosophy
OR: Odds ratio
CI: Confidence interval
EU: European Union
POMs: Prescription only medicines
FDA: Food and Drug Administration
USA: United States of America
UK: United Kingdom
GR: Greece
CY: Cyprus
MDD: Maximum daily dose
PRAC: Pharmacovigilance Risk Assessment Committee
