# Supplementary material for: Exploring public knowledge and perceptions regarding per os OTC pain-relieving medications: the case of paracetamol (acetaminophen)
Source: J Pharm Policy Pract. 2023 Jul 20;16:93. doi: 10.1186/s40545-023-00598-1 (PMC10360319; doi:10.1186/s40545-023-00598-1)
Supplement: Supplementary file 1 — Additional file 1: Appendix S1. A translated version of the given questionnaire (the original questionnaire was in Greek language). [file 40545_2023_598_MOESM1_ESM.doc]

**APPENDIX S1:**

A translated version of the given questionnaire (the original questionnaire was in Greek language).*

**QUESTIONNAIRE ON EXPLORING PUBLIC’S KNOWLEDGE AND PERCEPTIONS REGARDING PER OS OTC PAIN-RELIEVING MEDICATIONS: THE CASE OF PARACETAMOL (ACETAMINOPHEN)**

By completing this questionnaire you give your implied consent. The completion is on voluntary basis and anonymous.

**GENDER**: ……………………….

**AGE**: ……………………….

**LOCATION**: ………………………. **EDUCATIONAL BACKGROUND**

**A.** Middle School graduate

**B.** High School graduate

**C.** University graduate

**D.** Postgraduate or PhD

**E.** Healthcare professional

**QUESTIONNAIRE

1. Which of the following medications do you believe that it contains paracetamol as the main active compound?**

**A.** DEPON

**B.** PANADOL

**C.** PONSTAN

**D.** ASPIRIN

**E.** NIMM

**F.** VOLTAREN

**G.** APOTEL

**H.** BUSCOPAN PLUS

**I.** SOLPADEINE

**2. How often do you use paracetamol?**

**A.** Every day

**B.** Two times per week

**C.** Once per week

**D.** Once per month

**E.** Based on my needs

**3. For which reason did you use paracetamol previously?**

**A.** Headache

**B.** Arthralgia/abdominal pain

**C.** Cold and flu

**D.** Fever

**E.** Dizziness

**F.** Other (please specify): ……………………….

**4. What was the paracetamol dosage that was taken previously?**

**A.** One pill

**B.** 2–3 pills per day

**C.** One pill every day for 3–4 days

**D.** One pill every day for one week

**E.** One pill every day for more than one week

**5. During the last time you used paracetamol, who recommended it?**

**A.** My doctor

**B.** My doctor suggested it in the past

**C.** My pharmacist

**D.** A relative

**E.** A friend of mine

**6. Do you know what is the maximum daily allowed dose of paracetamol?**

**A.** 500 mg

**B.** 1 g

**C.** 3 g

**D.** 4 g

**E.** 5 g

**7. Where do you get information about paracetamol use?**

**A.** My friends and relatives

**B.** My doctor

**C.** My pharmacist

**D.** Internet (webpages)

**E.** Advertisements on TV/Internet

**8. How often do you use analgesics on your own initiative?**

**A.** Every day

**B.** 2–3 times per week

**C.** Once per week

**D.** Once per month

**E.** Other (please specify): ……………………….

**9. Do you consume alcohol after the use of paracetamol?**

**A.** Never

**B.** Yes, 2–3 hours following paracetamol dose

**C.** Yes, because it doesn’t matter

**10. For patients under chronic treatment only: Do you inform your doctor before paracetamol use?**

**A.** No

**B.** Sometimes I will call the doctor

**C.** Only when I visit the doctor

**D.** Never

**11. For patients under chronic treatment only: Do you inform your pharmacist about the medications taken for your chronic condition(s) before paracetamol use?**

**A.** No

**B.** Sometimes

**C.** Only if he/she asks

**D.** Always

**E.** I find it redundant

**12. Do you believe that chronic use of paracetamol can cause toxic side effects?**

**A.** No

**B.** Yes

**13. If paracetamol was freely available in the market (other than the pharmacy, e.g., supermarket, etc.), would you buy it from there?**

**A.** Yes

**B.** Yes, if I was in a hurry

**C.** In some cases, if I have already bought paracetamol from a pharmacy before

**D.** No, since I would like to have an expert advice

**E.** No, since I’m not sure if they have been stored appropriately

**14. After drinking alcohol, do you consume paracetamol-containing analgesics?**

**A.** Yes

**B.** No

* Footnote: The participant’s information sheet was given in a separate document.
